# Acute effects of motor-cognitive injury-prevention warm-ups on unplanned jump-landing performance in team-sport athletes: A randomized crossover trial

**DOI:** 10.1371/journal.pone.0360379

**Published:** 2026-10-09

**Authors:** Felix Laukhardt, Lutz Vogt, Thorben Hülsdünker, Tobias Engeroff, Winfried Banzer, David Groneberg, Florian Giesche

**Affiliations:** 1 Institute of Occupational, Social and Environmental Medicine, Goethe University, Frankfurt/Main, Germany; 2 Department of Sports Medicine and Exercise Physiology, Goethe University, Frankfurt/Main, Germany; 3 Department of Sport, LUNEX, Differdange, Luxembourg; 4 Luxembourg Health and Sport Science Research Institute, Differdange, Luxembourg; 5 Medical Department, Eintracht Frankfurt, Frankfurt/Main, Germany; Universidade de Aveiro Escola Superior de Saude de Aveiro, PORTUGAL

## Abstract

In team-sports, agility, perceptual-cognitive abilities, and executive functions are crucial for performance and injury prevention. Incorporating motor-cognitive demands into neuromuscular warm-ups may improve coping with cognitively demanding movement situations, requiring time-constrained decision-making and reactive responses. This randomized three-arm crossover study investigated the acute effects of the Prevent Injury and Enhance Performance (PEP) warm-up and two adapted motor-cognitive versions (PEP+; PEP-SKILLCOURT) on pre-planned (n = 6) and unplanned countermovement jump-landings, with the landing side cued only after take-off (n = 16). Eighteen team-sport athletes (age: 23 ± 3 years; height: 181 ± 11.4 cm; weight: 75.6 ± 10.4 kg; 5 females) completed the three warm-ups on separate days. PEP+ included cognitive exercises targeting visuomotor processing and executive functions. PEP-SKILLCOURT incorporated reactive agility exercises using the SKILLCOURT device. After each warm-up, participants performed the jump-landing task on a capacitive pressure plate. Landing performance was assessed using biomechanical and error-related outcomes, including peak vertical ground reaction force (pVGRF), time to pVGRF, center of pressure path length (CoP), time to stabilization (TTS), standing errors, and decision errors. Linear and generalized mixed models were used to analyze biomechanical outcomes and error counts, respectively, adjusting for flight time. No significant carryover effects occurred (p > .05). Landing condition significantly affected pVGRF and CoP (−4 to +17%, p = .003–.004) as well as error rates (p = .002). Post hoc tests indicated lower pVGRF, greater CoP fluctuations (p = .041 to <.001), and more errors (p = .037 to <.001) during unplanned landings. After Holm adjustment, no significant treatment or treatment × landing condition interaction effects remained. However, exploratory trends suggested lower landing loads and reduced CoP fluctuations after PEP+. Unplanned conditions resulted in poorer landing control and more erroneous landings, indicating reduced task performance. Larger trials are needed to confirm the exploratory trends favoring PEP+ and examine joint-specific biomechanics relevant to injury risk.

## Introduction

Team-sports such as football (soccer) or basketball show the highest injury prevalence across all sports [1]. A large proportion of these injuries affect the knee or ankle ligaments [2], often leading to prolonged rehabilitation, increased reinjury rates, and shortened career lengths [3]. Most severe lower limb injuries, such as anterior cruciate ligament (ACL) ruptures, occur without direct contact [4], typically during failed single-leg landings, cutting or deceleration maneuvers performed in response to sudden or unforeseen events (e.g., ball or player movement, deceptive or unexpected opponent actions) under substantial time-constraints [5,6]. The ability to rapidly adjust movement plans and actions in response to such unexpected and unplanned changes on the field, defined as agility [7], is thus crucial for both injury prevention and performance in team-sports [7,8].

Recent research aimed to replicate such unplanned tasks using single-leg landings or cutting maneuvers performed in response to visual cues presented shortly before ground contact (approximately 300–600 ms), indicating the landing leg or movement direction. Specifically, these studies suggest that unplanned conditions result in altered global biomechanical indicators commonly used as functional surrogate markers of dynamic landing control (e.g., greater center of pressure fluctuations; CoP) and load absorption (e.g., higher and faster peak vertical ground reaction forces; pVGRF [9–11]). Unplanned movement tasks have also been associated with a greater number of erroneous landings (e.g., incorrect leg placement or loss of balance) [9,10,12], as well as alterations in joint-specific biomechanics at the knee and ankle (e.g., reduced knee flexion angles or increased abduction moments), which may reflect an increased injury risk, compared with pre-planned conditions that allow for sufficient feedforward control [13–15].

Dealing with unplanned demands requires substantial cognitive resources. Cross-sectional evidence indicates that higher perceptual-cognitive performance (e.g., visuomotor reaction speed) and executive functions (e.g., working memory, cognitive flexibility, and inhibitory control [16]) are associated with improved dynamic movement control and more favorable knee biomechanics (e.g., increased knee flexion angles or lower abduction moments) under unplanned conditions [17–20]. Prospective evidence further suggests that lower pre-season cognitive performance (e.g., slower visuomotor reaction speed) is associated with an increased risk of non-contact injuries (e.g., ACL-injuries) during the playing season [17,21]. Beyond injury prevention, higher cognitive performance, particularly executive functions has also been linked to greater playing performance and expertise in youth team-sport athletes [22,23].

Current research suggests that incorporating reactive agility drills (i.e., multidirectional whole-body movements in response to unplanned visual stimuli [7]) and cognitive exercises (e.g., visuomotor reaction speed, executive functions) into warm-up protocols may enhance athletes’ ability to cope with complex motor-cognitive demands, such as time-pressured decision-making during movement [24–26]. In contrast, widely used programs such as the Prevent Injury and Enhance Performance (PEP) program [27], do not include such components. Consistent with this, a recent study reported that the PEP program improved single-leg landing stability only under pre-planned conditions (i.e., without time-constrained decision-making), but not during unplanned tasks [11].

The aim of the present study was to examine how incorporating specific cognitive and agility demands into the standard PEP warm-up acutely affects jump-landing performance, assessed via global biomechanical and error-related outcomes, reflecting dynamic landing control and impact absorption, during pre-planned and unplanned single-leg jump-landings in amateur team-sport athletes. We hypothesized that (1) performance would be poorer under unplanned compared with pre-planned conditions, (2) performance would improve following the motor-cognitive adapted PEP programs compared with the standard PEP program, and (3) there would be a treatment (PEP program) × landing condition interaction, with the greatest performance benefits observed under unplanned conditions, as the adapted programs impose greater cognitive demands and may therefore better prepare athletes for such situations.

## Materials and methods

### Study design and ethical aspects

We conducted a three-arm randomized crossover study. Participants were randomly assigned by the investigators to one of six intervention sequences using sequentially numbered allocation. A flow diagram illustrating participant progression through the crossover design is shown in Fig 1. No separate full-length trial protocol was prepared or published for this study. However, the protocol was based on a previous similar crossover study conducted by our research group [28]. The present study was conducted under the same trial registration as a previous study from our research group (DRKS00016942; 24 May 2019; www.drks.de). Thus, the registration predated the present study and was not prospectively updated to reflect the protocol reported here. The present study included specific deviations from the registered protocol, none of which were introduced after examination of the outcome data. These deviations included restricting the sample to healthy participants and adding a motor-cognitive warm-up intervention, thereby expanding the design to a three-arm crossover trial. All deviations from the originally registered protocol are documented in the CONSORT flow diagram and S1 Table. S1 File includes a CONSORT checklist about this randomized trial. S2 File contains the complete anonymized dataset underlying the reported analyses.

**Fig 1:**
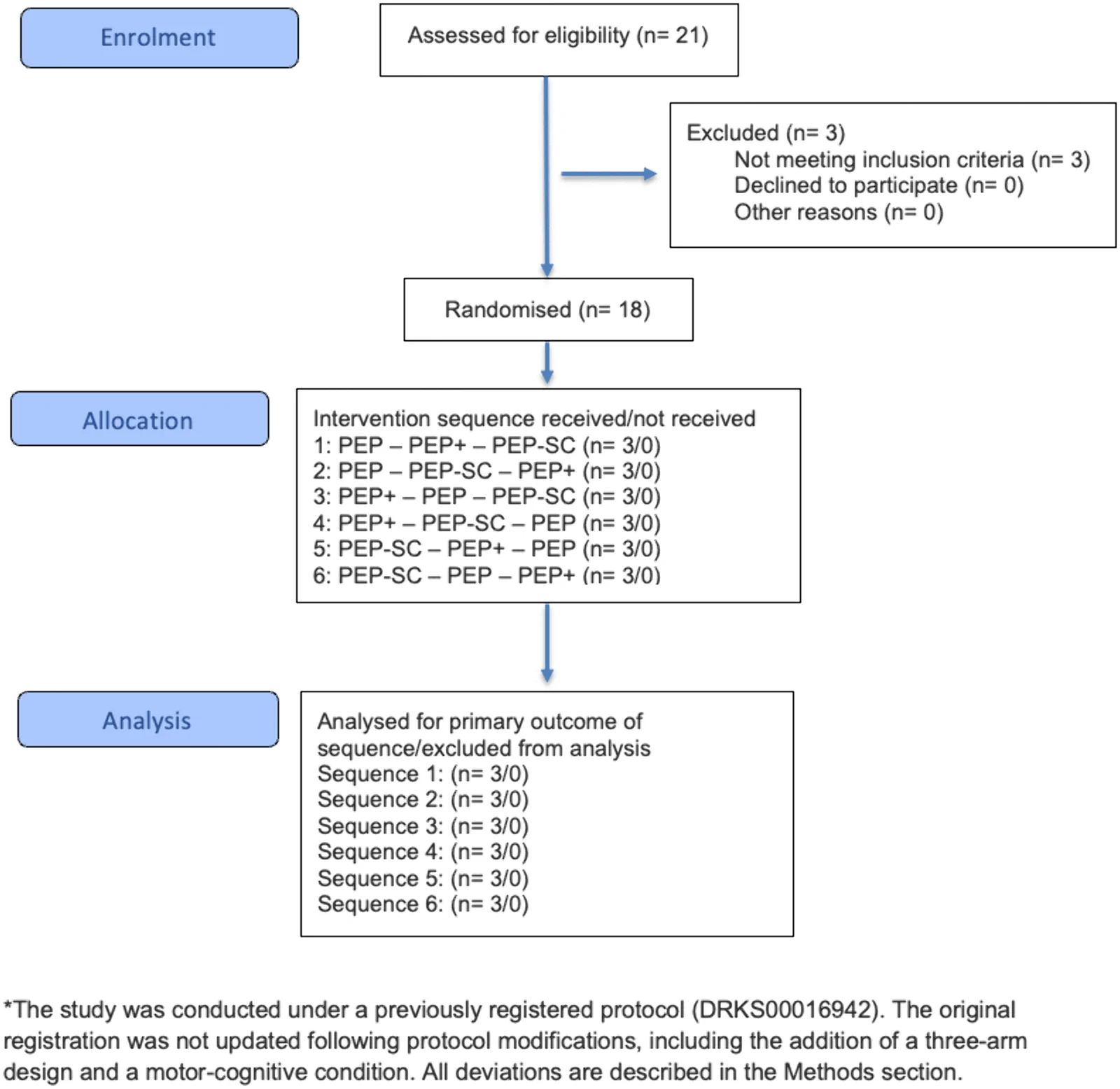
CONSORT 2025 flow diagram. Flow diagram illustrating participant progression through the phases of a randomized crossover trial with six different treatment sequences, including enrolment, intervention allocation, and data analysis.

The investigation was approved by the local Ethics Committee (number 2017−27) and carried out according to the ethical standards set by the Declaration of Helsinki (Version Fortaleza, 2013). Each participant signed a written informed consent form before participating in the study.

### Participants

Recruitment was performed at local sports clubs and the university sports campus using flyers, e-mails, social media, and personal addressing. Inclusion criteria were (1) participation in team-sport (e.g., football, basketball, volleyball), (2) age between 18 and 30 years, and (3) minimum countermovement jump (CMJ) height of 30 cm (corresponding to a flight time of ~500 ms). Participants were excluded if they (1) had injuries to the lower extremities or head (e.g., concussion) within the past six months, (2) reported an acute or chronic disease affecting performance or quality of life, (3) used mind- or perception-altering substances, or (4) reported muscle soreness. Participants were instructed to refrain from intensive physical activity and from consuming drugs (e.g., alcohol, cigarettes, or other substances) on the day before testing.

The required sample size was calculated using G*Power (version 3.1.9.6; Heinrich Heine University Düsseldorf, Germany) based on effect size estimates from a comparable two-arm crossover study using a repeated-measures ANOVA [11]. Similarly, the previous study investigated the acute effects of warm-up interventions on jump-landing performance during pre-planned and unplanned conditions in healthy team-sport athletes and reported large effects of the standard PEP warm-up compared with a cycling ergometer warm-up on time to pVGRF and CoP in the pre-planned condition (d = 1.27–1.29, corresponding to f ~ 0.64). As no treatment × landing condition interaction effects were reported in the previous trial and the present study included three warm-up interventions (rather than two in the previous trial), a more conservative effect size (f = 0.40) was assumed. Because the primary analyses were planned using a linear mixed model framework, this sample size calculation based on the previous study [11] was used as a pragmatic approximation. With α = 0.05, power = 0.80, r = 0.5, and ε = 0.75, the required sample size was n = 15, which was increased to 18 to account for a potential 20% dropout rate. However, as the sample size calculation was based on a different study design, the present trial should be regarded as exploratory and may be underpowered to detect potential treatment × landing condition interactions and smaller treatment effects. All participants were included in the analysis.

### Experimental setup

The three warm-up interventions, including the standard PEP program and two motor-cognitively adapted versions (PEP+, and PEP-SKILLCOURT; SC; as described in detail below), were conducted on three separate test days at the same time of day, with a washout period of at least seven days between sessions according to previous trials [11,28]. On each test day, participants completed one of the three warm-up interventions in random order. To assess potential physical or mental fatigue effects, maximum CMJ height and cognitive performance (pen-paper-Trail-Making-Test; TMT) were measured before and after each warm-up. Participants were blinded to the intervention order and were informed on the day of testing which warm-up they would perform. Each test day began with 30 s of jumping jacks, followed by a short jump-landing task and warm-up familiarization. The assigned warm-up was then performed. The actual jump-landing performance was assessed immediately after each warm-up. On the first test day, personal and anthropometric data were additionally collected. The data collection took place from May to August 2023.

### Interventions

All participants completed the standard PEP protocol [27] according to a slightly adapted version by Friebe et al. (2022) [11]. The warm-up protocol consisted of 12 exercises across four sections (general warm-up, strengthening, plyometrics and agility/change of direction). This version deviates from the original protocol insofar that it omits static stretching and “Russian Hamstring” exercise, as these have been shown to negatively influence reactive and explosive strength performance [29]. In addition, a minor modification was made to the jumping tasks by combining forward/backward and lateral jumps within sets using both clockwise and counter-clockwise sequences, rather than performing these sequences separately, to ensure consistency with the adapted PEP versions. Further details are provided in Table 1, and the detailed experimental setup for all three warm-up programs is depicted in Fig 2.

**Table 1:** Characteristics of the warm-up programs PEP, PEP+, and PEP-SC, including exercises, exercise goals, and duration.

	Category	Exercise	Goal	Duration
**PEP**	General warm-up	a) Jogging b) Shuttle Run c) Backward running	General preparation of the athlete	30s per exercise
	Strengthening	a) Lunges b) Single-legged calf raises	Activation and strengthening of the leg muscles Knee and ankle control	a) 60s b) 30s per leg
	Plyometrics	a) Lateral and forward/backward two-legged jumps b) Lateral single-legged jumps c) Vertical jumps headers d) Scissor jumps	Increase power, strength and speed Practicing landing technique Knee control	a) 2x 30s, 15s break b)-d) 30s (per leg)
	Agility	a) Forward run with 3 step decelerations b) Lateral cuttings and diagonal run c) Bounding run	Increase dynamic stability of the ankle/knee/hip complex	60s per exercise ***10 min.***
**PEP+**	General warm-up	a) Jogging b) Shuttle Run c) Backward running	General preparation of the athlete	30s per exercise
	Strengthening	a) Lunges **+ Digit span task forward and** **backward** b) Single-legged calf raises **+ Stroop word color** **interference test**	Activation and strengthening of the leg muscles Knee and ankle control **Activation of working memory and executive functions**	a) 60s b) 30s per leg
	Plyometrics	a) Lateral and forward/backward two-legged jumps **+ Direction instructions** b) Lateral single-legged jumps **+ Go/No-Go-Task (Fitlight)** c) Vertical jumps headers d) Scissor jumps	Increase power, strength and speed Practicing landing technique Knee control **Enhance decision-making speed and inhibitory control**	a) 2x 30s, 15s break b)-d) 30s (per leg)
	Agility	a) Forward run with 3 step decelerations **+ Reaction (Fitlights)** b) Lateral cuttings and diagonal run **+ Reaction (Fitlights)** c) Bounding run	Increase dynamic stability of the ankle/knee/hip complex **Enhance reactive agility and decision-making**	60s per exercise ***10 min.***
**PEP-SC**	General warm-up	a) Jogging b) Shuttle Run c) Backward running	General preparation of the athlete	30s per exercise
	Strengthening	a) Lunges b) Single-legged calf raises	Activation and strengthening of the leg muscles Knee and ankle control	a) 60s b) 30s per leg
	Plyometrics	a) Lateral and forward/backward two-legged jumps b) Lateral single-legged jumps c) Vertical jumps headers d) Scissor jumps	Increase power, strength and speed Practicing landing technique Knee control	a) 2x 30s, 15s break b)-d) 30s (per leg)
	Agility	**a) Dual-Task Agility** **(SKILLCOURT)** **b) 3x Random Run 25m** **(SKILLCOURT)**	**Enhancement of reactive agility, spatial awareness inhibitory control and decision-making**	**a) 11x 10s** **b) 3x 10-15s** **10 min.**

**Fig 2:**
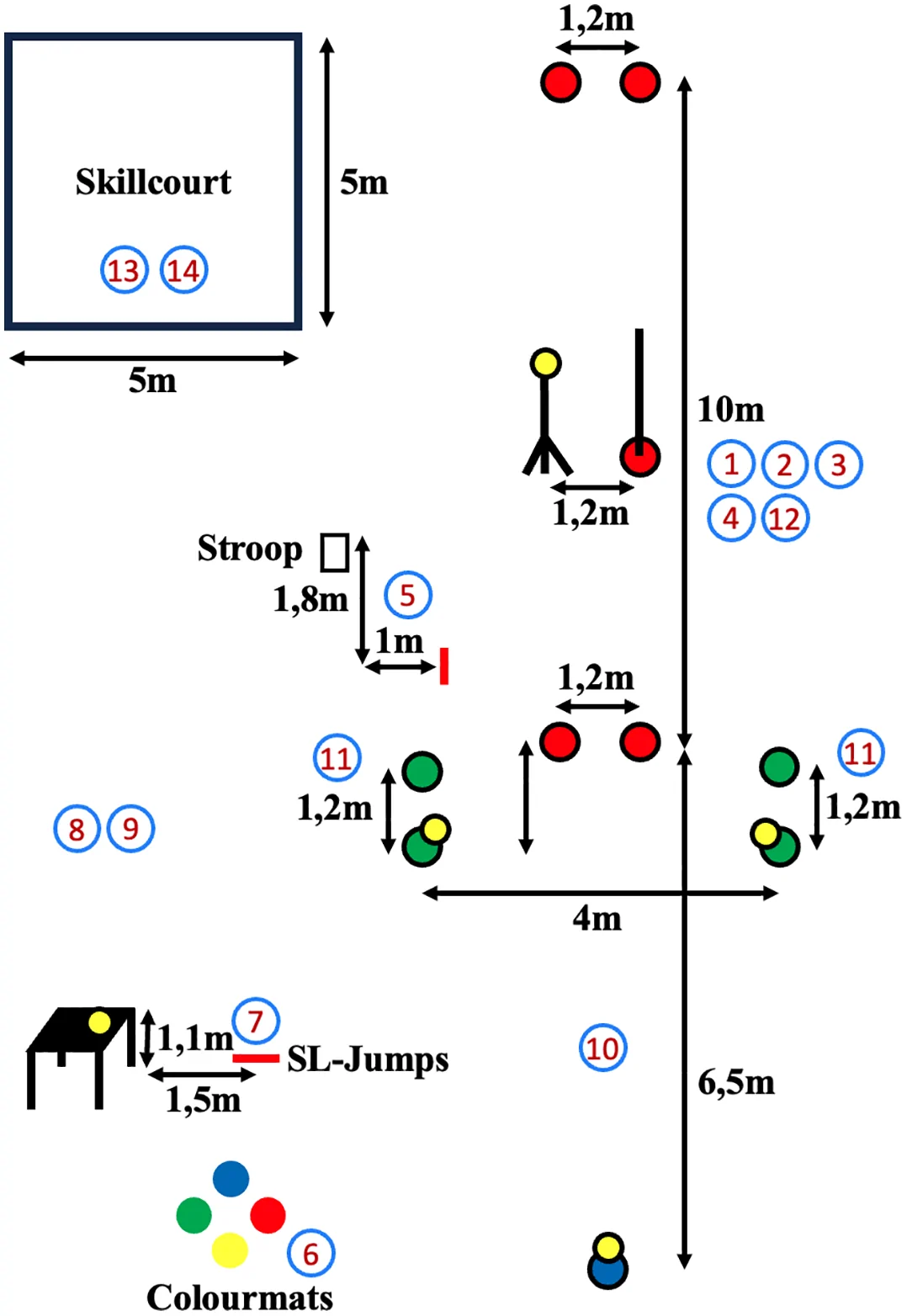
Experimental setup. Experimental setup showing the station layout, inter-station distances, and exercise allocation across the different warm-up programs. Yellow circles indicate Fitlights (FITLIGHT, DynamicEye, Cologne, Germany), and larger colored circles indicate cones. Numbers refer to exercise stations: (1) jogging, (2) shuttle run, (3) backward running, (4) walking lunges with a digit span task (recall of number sequences forward/backward; starting with three digits and increasing by one digit after two correctly repeated sequences), (5) single-leg calf raises with Stroop word-color interference task (30 s per leg), (6) two-legged lateral and forward/backward jumps with color/direction instructions: participants started in the center of four rubber color mats (30 cm diameter) and responded to verbal cues indicating either direction or color (e.g., direction, direction, color, color, direction, etc.); instructions were given upon landing outside the mats, (7) single-leg lateral jumps over a line using a Go/No-Go-Task with Fitlights (jump on green, hold on red; lights appear randomly for 1.5-2.5 s every 3-5 s), (8) vertical jump headers, (9) scissor jumps, (10) forward run with three-step deceleration and visual stop signal (red) via Fitlight (blue cone), (11) lateral cutting and diagonal running with directional cues (left/right) via Fitlights (green cones), (12) bounding run, (13) dual-task agility on the SKILLCOURT involving ball-tracking and reactive runs toward randomly displayed target fields, (14) 25 m random run on the SKILLCOURT involving reactive multidirectional runs toward randomly displayed target fields.

For the PEP+ protocol, specific cognitive tasks were added to selected exercises. Established cognitive tasks (digit span test, Stroop Color and Word Test, Go/No-Go task, and reaction speed tasks) were used to target visual perception, psychomotor reaction speed, and executive functions, which are considered relevant for performance and injury prevention in team-sports [5,6,22,23,30]. During PEP+, participants were exposed to additional cognitive load for 50% of the session duration. A detailed overview of the PEP+ exercises is provided in Table 1.

The PEP-SC protocol was adapted by replacing the agility/change-of-direction section of the original PEP with agility exercises (i.e., unplanned or reactive changes of direction/runs) using the SKILLCOURT (SKILLCOURT GmbH, Schweinfurt, Germany; Fig 3). This component included a reactive agility exercise (Random Run 25 m) and a dual-task exercise combining an agility task with an integrated 3D multiple object tracking task requiring dual-/multi-tasking (exercise details are shown in Fig 4). The SKILLCOURT system presents motor-cognitive tasks on a 65-inch screen and tracks participants’ position on a 5 × 5 m rubber mat court using a LiDAR-based system (for technical details, see Friebe et al. (2023) [31]). The remaining components of the protocol were unchanged. During PEP-SC, participants performed agility exercises (single- and dual-task) for approximately 25% of the session duration on the SKILLCOURT system. All three warm-up protocols shared an identical overall structure (sections) with matched exercise duration across components. However, in PEP-SC, these exercises were implemented to increase reactive agility and motor-cognitive demands.

**Fig 3:**
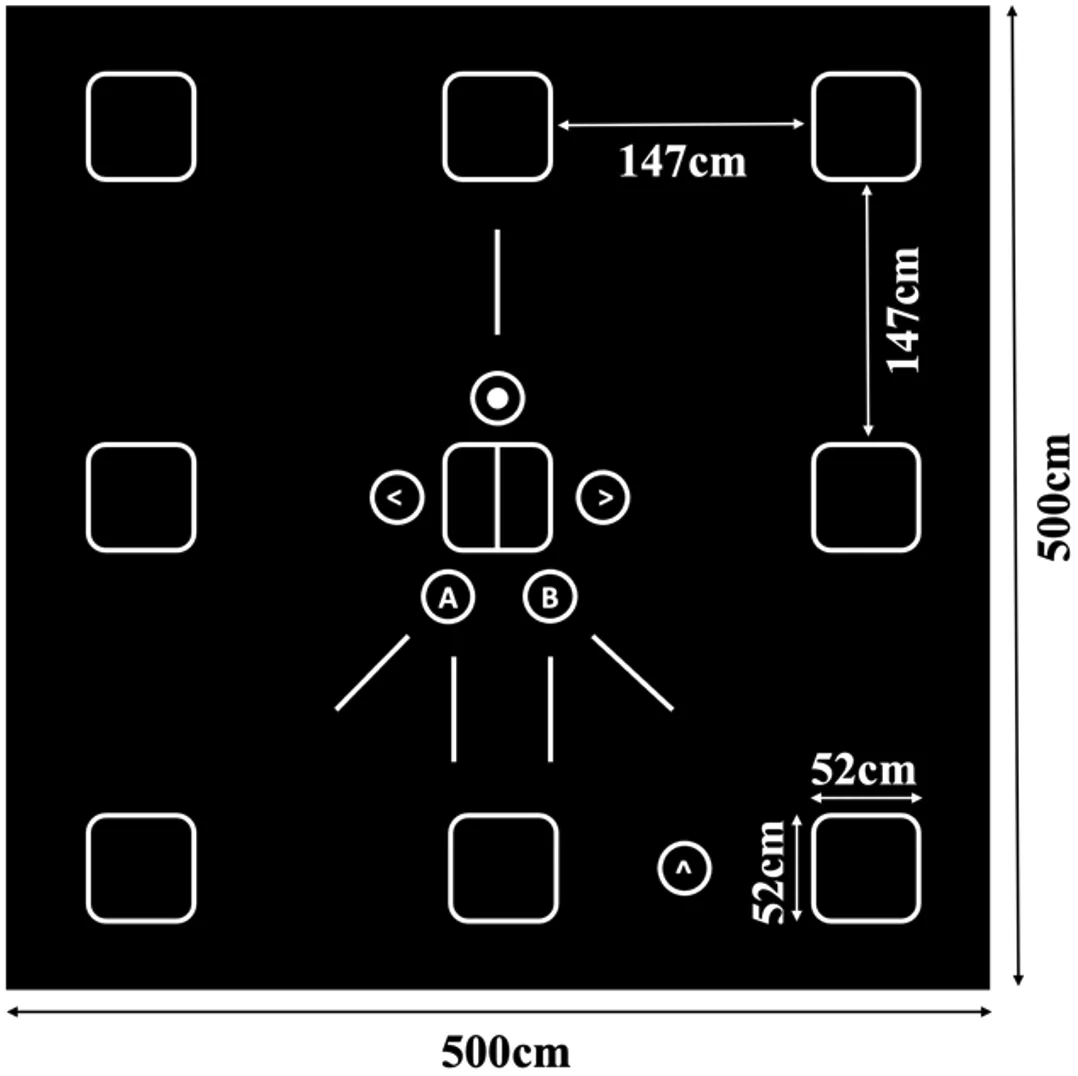
Schematic illustration of the SKILLCOURT with size specification.

**Fig 4:**
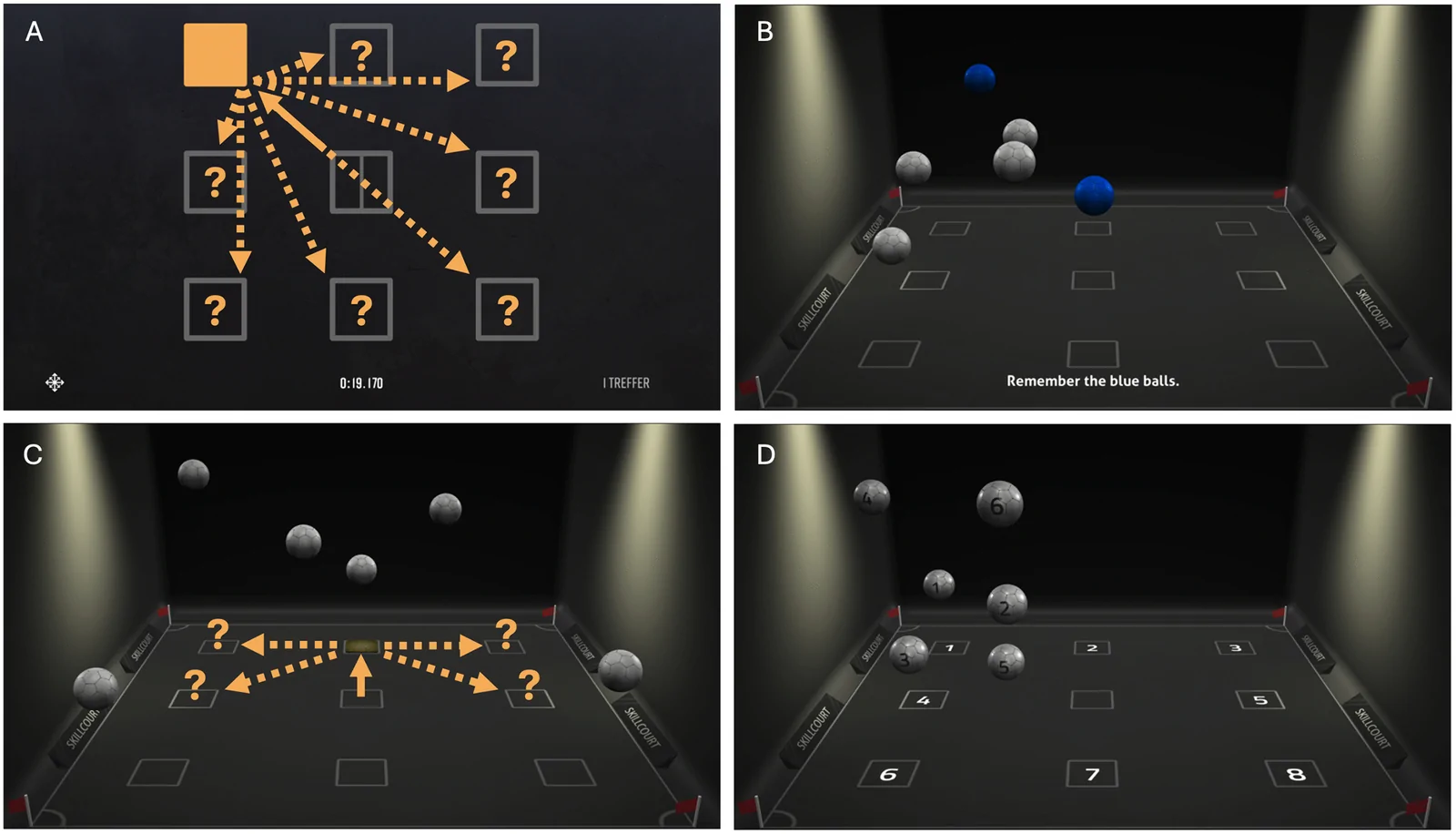
Exemplary illustration of SKILLCOURT-tasks. (A) Random Run 25 m: Participants started in the center and ran to the target field indicated in yellow, then proceeded to the next yellow target field according to a random sequence until a total distance of 25 m was reached. (B) Dual-task start screen: Participants were instructed to memorize the blue balls, which turned white once movement began. (C) Dual-task execution: Participants tracked the blue balls while simultaneously running to the yellow target fields, again in a randomized order. (D) Dual-task response selection: Once the balls stopped, each ball was assigned a number; participants were instructed to step onto the corresponding numbered fields that matched the balls shown in blue on the start screen.

Before the start of each intervention, the exercises of the respective warm-up protocol were explained and demonstrated. Participants were allowed to ask questions and briefly practice the exercises to ensure correct execution. Familiarization procedures (instruction, demonstration, and practice trials) were identical across all warm-up conditions and of comparable duration. During interventions, movement quality was monitored by the test instructor and corrected if necessary. After completion of each intervention, participants rested in a seated position until perceived exertion decreased to a Borg rating of 10 (low exertion), with a minimum rest period of three minutes to prevent potential fatigue-related confounding. Afterwards, the actual jump-landing-task assessment was performed.

### Jump-landing-task

The participants completed 22 countermovement jumps (CMJ) with pre-planned (*n* = 6) or unplanned (*n* = 16) single-leg landings (randomized order, landing limb evenly distributed) on a capacitative pressure plate. The unplanned condition was subdivided into eight expectation-consistent (landing leg did not change after take-off) and eight expectation-inconsistent trials (landing leg changed after take-off; see detailed description below).

Prior to each jump, the required landing leg (left or right footprint) was shown on a presentation slide (Microsoft PowerPoint 2010) displayed on a 17-inch screen positioned 2.5 m in front of the participants (Fig 5). Participants were instructed to land on the indicated leg unless the displayed landing side changed after take-off. Slide transitions (left/right footprint) were triggered by a USB foot switch (PC Sensor USB foot switch Keyboard; China) positioned under the jump platform and connected to a laptop (Fig 5). When the participant left the platform, the switch was released, prompting the displayed landing side to change with a latency of approximately 100 ms (kinovea software, version 2023.1.2; 60 frames/s). Participants were instructed to land on the specified leg, regain a stable position as quickly as possible, and maintain it for 10 s (hands on hips and eyes focused on a fixation cross at eye level). Trials were performed consecutively.

**Fig 5:**
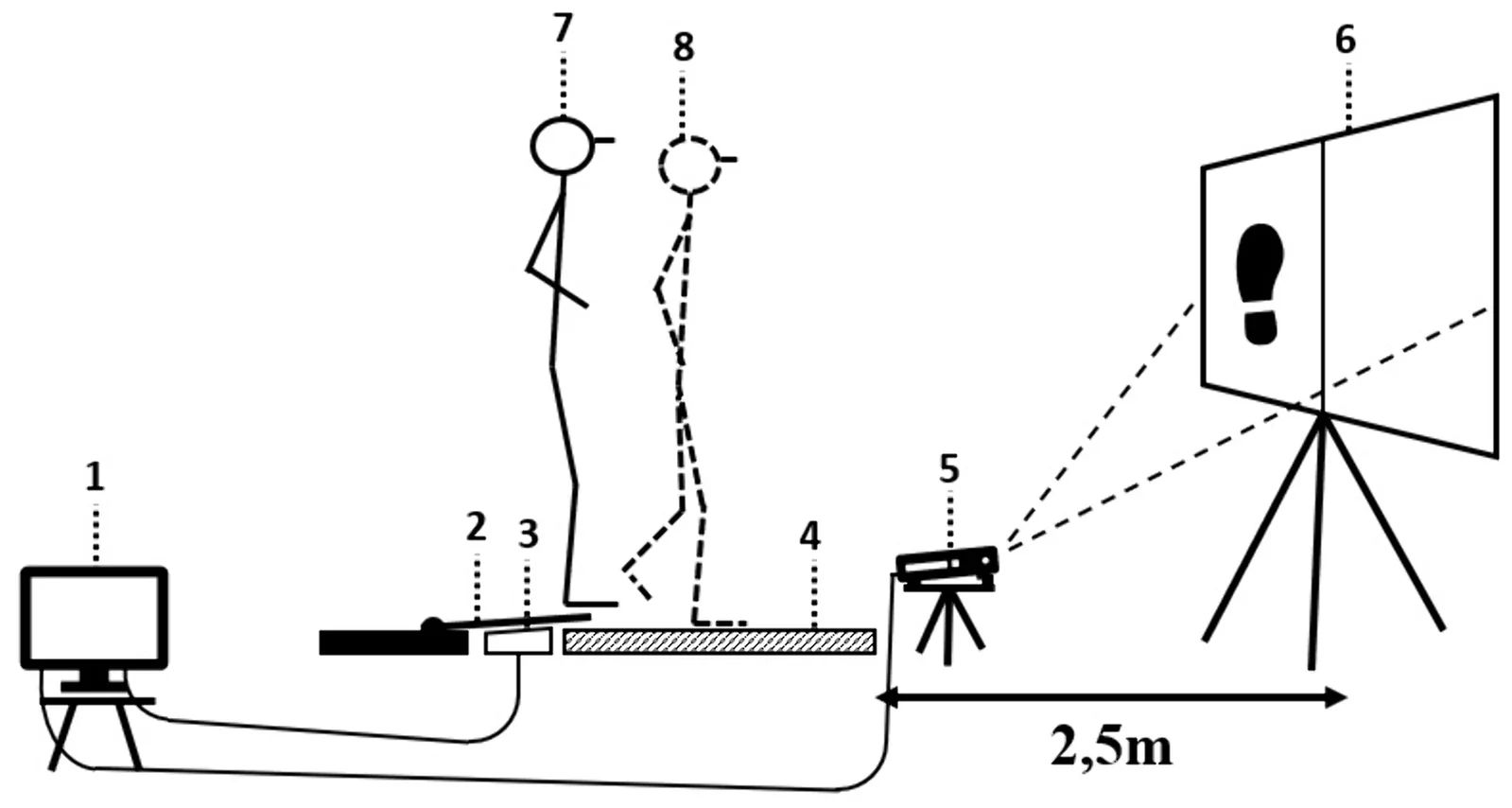
Setup of the jump-landing task. (1) computer with Microsoft PowerPoint slides, (2) board for operating the switch, (3) switch, (4) pressure plate, (5) beamer, (6) screen, (7) participant before the jump, and (8) participant after landing.

For the pre-planned trials, participants were informed prior to take-off that the indicated landing leg would remain unchanged, allowing execution without any additional cognitive demands (i.e., no time-constrained decision-making). In the unplanned condition, participants were also instructed to land on the initially displayed leg, but were not informed whether the indicated landing leg would change after take-off, requiring responses to a potentially changing stimulus during the jump (i.e., time-constrained decision-making). In 8 of the 16 unplanned trials (expectation-consistent condition), the indicated landing leg remained unchanged, allowing execution of the pre-planned motor pattern. In the remaining 8 trials (expectation-inconsistent condition), the landing side changed after take-off, requiring inhibition of the initial motor plan and rapid movement adjustment. Due to randomization, this requirement was unpredictable. The conditions represent a progressive increase in motor-cognitive demands and stimulus uncertainty, ranging from pre-planned (high certainty, no time-constrained decision-making), to unplanned expectation-consistent (high uncertainty without the need for reactive movement adjustment under time-constraints), and finally to expectation-inconsistent trials (high uncertainty with the need for reactive movement adjustments under time constraints). This setup, consistent with previous work [32], was designed to mimic on-field situations in which team-sport athletes may either execute pre-planned movements and actions when outcomes are predictable (expectation-consistent condition) or rapidly adapt their strategies when unexpected changes occur under substantial time pressure (expectation-inconsistent condition).

Included participants were required to achieve a countermovement jump height of at least 30 cm, corresponding to a flight time of approximately 500 ms. After accounting for the latency of the button switch, this resulted in an available response time of ~400 ms during the unplanned condition, consistent with previous trials [33,34]. Participants were instructed to jump as high as possible in all trials to ensure sufficient response time and comparability between conditions. No minimum number of successful trials per condition was predefined, and no participants were excluded based on the number of successful trials.

Before the warm-up interventions on each test day, a short familiarization session with the jump-landing task was conducted, including instructions and demonstration by an experimenter, followed by two to three practice trials per condition.

### Outcomes

A capacitive pressure plate (Zebris PDM-S, V1.2.1; Zebris Medical GmbH, Isny, Germany; 60 Hz, 52 × 38 cm) was used to assess biomechanical landing control. The pressure plate has been shown to be a valid and reliable tool for the assessment of balance and dynamic postural stability in both healthy and clinical populations [35,36].

Jump-landing performance was assessed using multiple biomechanical outcomes reflecting dynamic landing control and impact load absorption. These variables are widely used in the literature as global indicators of dynamic landing performance during pre-planned and unplanned movement tasks in healthy and ACL-reconstructed individuals and may therefore be interpreted as functional surrogates of overall movement control, as reported in previous studies [10,24,37–39], rather than direct assessments of joint-specific biomechanics (e.g., joint angles and moments). Nevertheless, these measures may still be relevant for injury risk, as high impact forces and impaired dynamic landing control have been discussed as potential contributors to non-contact injuries (e.g., ACL injuries) [37–41]. The following parameters were determined: (1) peak vertical ground reaction force (pVGRF; N/kg), (2) time to pVGRF (ms), (3) center of pressure path length (CoP; mm), defined as the average displacement in the anterior-posterior and medio-lateral directions [42] during the first 2.5 s after landings [43], (4), and time to stabilization (TTS; s), defined as the time required to regain a stable stance within a 10-s period after landing [44]. TTS was calculated according to Colby et al. (1999) [45] and Wikstrom et al. (2005) [44] and has been shown to demonstrate moderate to high reliability [46].

Additionally, standing and decision error counts were recorded across jump-landing trials as qualitative performance measures based on previous work [9,11,28]. Standing errors were defined as landings performed on the correct leg in which the free leg or any other body part touched the ground, or cases where the landing foot left the platform during the 10-s recording period. Decision errors were defined as landings on the wrong foot or on both feet in the unplanned condition, reflecting an unsuccessful adjustment of the required motor plan and action within the available response time during the jump. Landing stability outcomes (CoP and TTS) were analyzed for successful trials only, whereas ground reaction forces were analyzed across all trials. In addition, flight time (jump height/available response time) was recorded for each trial to account for potential confounding effects on quantitative and qualitative jump-landing performance measures according to previous work [9]. The participants’ level of work- and sports-related activities were assessed by the Tegner Activity Scale [47].

Finally, to assess potential confounding effects of fatigue on jump-landing performance, three CMJs (flight time; ms) were performed on the pressure plate pre- and post-intervention as an indirect measure of lower-limb concentric strength and explosive power [48]. In addition, the Trail-Making-Test (Versions A and B; completion time; s) was administered pre- and post-intervention to assess the potential effects on cognitive processing and executive functions [49]. To minimize learning effects across the three test days, different test versions were used in random order.

### Statistical analysis

After an initial plausibility check of the data, linear mixed-effects models were fitted for each biomechanical outcome using Satterthwaite’s approximation for degrees of freedom to estimate fixed effects of landing condition (three jump-landing conditions), treatment (three warm-up interventions), and their interaction. Models included random intercepts for participants to account for within-participant repeated measures and were adjusted for average flight time across trials and potential period effects (i.e., test day-related learning or practice effects). Carryover (previous treatment) and sequence effects (based on the six treatment orders) were assessed separately while excluding the period factor to ensure model identifiability. Data were analyzed in long format, with trials averaged within each participant for each treatment and landing condition prior analysis, resulting in one observation per participant for each treatment and landing condition. Treatment, sequence, and carryover effects were included as fixed factors.

Model assumptions were evaluated using residual diagnostics (Q–Q plots and residuals vs. fitted values for normality and homoscedasticity), inspection of random effects distributions, and variance inflation factors (VIF) to assess multicollinearity. Influential observations were identified using Cook’s distance (threshold: 4/n), and sensitivity analyses were conducted excluding influential cases. Outliers were inspected using boxplots and retained, as their exclusion did not meaningfully affect fixed-effect estimates.

Estimated marginal means derived from the mixed models were used to obtain model-adjusted descriptive statistics (mean, standard error, degrees of freedom, and 95% confidence intervals) and to visualize interaction effects. To control for multiple testing, Holm adjustments were applied across all outcomes for fixed effects (condition, treatment, and their interaction) and post hoc comparisons. Marginal and conditional R^2^ values were computed to quantify variance explained by fixed effects alone and by the full mixed-effects model (including random intercepts), respectively. Additional covariates (age, sex, and Tegner activity level) were included in separate sensitivity analyses to assess robustness but were not part of the primary models.

Error-related outcomes were analyzed using generalized linear mixed-effects models with a negative binomial distribution and the same fixed and random effects structure as the linear mixed effects models described above. Zero counts were observed but were considered limited (standing errors: three participants showed zero errors across all three treatments; decision errors: one participant showed zero errors across all treatments). Sensitivity analyses using Poisson models yielded comparable results. Pre–post differences in CMJ height and Trail-Making-Test completion time were analyzed using paired Wilcoxon signed-rank tests. Adverse events (e.g., injuries) occurring during data collection were recorded. Statistical analyses were conducted in Jamovi (version 2.7.4), and the significance level was set at p < .05.

## Results

### Participants

Twenty-one athletes were initially recruited for the study. Due to not reaching the minimal jump height of 30 cm, three female athletes did not meet the inclusion criteria. Following this, eighteen adult amateur team-sport athletes (age: 23.1 ± 2.97 years; height: 181 ± 11.4 cm; weight: 75.6 ± 10.4 kg; Tegner activity score of 8 ± 2.0; 44% football, 33% basketball, 11% American football, 6% tennis, 6% volleyball; 5 females) were included. No dropout and no adverse events occurred.

### Carryover and sequence effects

No carryover effects from the previous treatment were detected for any biomechanical or error-related outcomes (*p* > .05). Similarly, no significant sequence effects were observed for any outcome (p > .05).

### Main fixed effects for biomechanical outcomes

Significant main effects of landing condition were observed for pVGRF and CoP (Tables 2 and 3). Holm-adjusted post hoc tests indicated lower pVGRF in the unplanned expectation-inconsistent condition compared with the pre-planned condition. CoP fluctuations were higher in both unplanned conditions and greater in the expectation-inconsistent compared with the expectation-consistent condition.

**Table 2:** Fixed effects from the linear mixed model for pVGRF, Time to pVGRF, CoP, TTS (adjusted for period and average flight); Holm-adjusted for multiple testing.

pVGRF	F	df1	df2	p	p Holm
Landing condition	7.48	2	133	<.001	**.003** ^**1**^
Treatment	1.41	2	134	.249	.498
Treatment × landing condition	0.87	4	133	.483	.712
Period	2.00	2	134	.139	
*R*^*2*^ *marginal: 0.153, p < .001; R*^*2*^ *conditional: 0.886, p < .001*					
**Time to pVGRF**					
Landing condition	0.40	2	133.6	.671	1.000
Treatment	4.23	2	134.4	.017	.068
Treatment × landing condition	1.84	4	133.7	.126	.504
Period	1.17	2	133.8	.315	
*R*^*2*^ *marginal: 0.052, p = .031; R*^*2*^ *conditional: 0.774, p < .001*					
**CoP**					
Landing condition	17.10	2	132.8	<.001	**.004** ^**2**^
Treatment	2.46	2	133.6	.089	.267
Treatment × landing condition	1.41	4	132.9	.234	.702
Period	0.74	2	133.0	.481	
*R*^*2*^ *marginal: 0.119, p < .001; R*^*2*^ *conditional: 0.589, p < .001*					
**TTS**					
Landing condition	0.69	2	132.5	.502	1.000
Treatment	1.16	2	133.3	.318	.498
Treatment × landing condition	1.11	4	132.7	.356	.712
Period	0.94	2	132.7	.395	
*R*^*2*^ *marginal: 0.067, p = .291; R*^*2*^ *conditional: 0.652, p < .001*					

***Post hoc comparisons:***

^*1*^ *PP vs. UN-EC: t = 2,25, df = 133, p = .026, p Holm = .052*

^*1*^ *PP vs. UN-EI: t = 3.85, df = 133, p = < .001, p Holm =* ***< .001***

^*1*^ *UN-EC vs. UN-EI: t = 1.60, df = 133, p = .112, p Holm = .112*

^*2*^ *PP – UN-EC: t = −2.06, df = 133, p = .041, p Holm =* ***.041***

^*2*^ *PP – UN-EI: t = −5.77, df = 133, p = < .001, p Holm =* ***< .001***

^*2*^ *UN-EC – UN-EI: t = −3.72, df = 133, p = < .001, p Holm* ***< .001***

Abbreviation: pVGRF = peak vertical ground reaction force; CoP = center of pressure path length; TTS = time to stabilization; PP = pre-planned; UN-EC = unplanned expectation-consistent; UN-EI = unplanned expectation-inconsistent

**Table 3:** Estimated marginal means for pVGRF, Time to pVGRF, CoP, TTS by Condition and Treatment (adjusted for period and average flight time).

pVGRF (N/kg)	Level	Mean	SE	df	95%-CI
Landing condition	PP	41.5	1.33	18.1	38.7; 44.3
	UN-EC	40.6	1.33	18.1	37.8; 43.4
	UN-EI	39.9	1.33	18.1	37.1; 42.7
Treatment	PEP	40.7	1.33	18.1	37.9; 43.5
	PEP+	40.3	1.33	18.1	37.5; 43.1
	PEP-SC	41.0	1.33	18.1	38.2; 43.8
**Time to pVGRF (ms)**					
Landing condition	PP	60.3	2.36	19.2	55.4; 65.2
	UN-EC	59.4	2.36	19.2	54.5; 64.4
	UN-EI	60.2	2.36	19.2	55.2; 65.1
Treatment	PEP	59.7	2.36	19.2	54.8; 64.6
	PEP+	61.6	2.36	19.2	56.6; 66.5
	PEP-SC	58.6	2.36	19.2	53.7; 63.6
**CoP (mm)**					
Landing condition	PP	503	21.8	22.8	458; 549
	UN-EC	534	21.8	22.8	489; 579
	UN-EI	589	21.9	23.0	543; 634
Treatment	PEP	556	21.8	22.8	511; 601
	PEP+	524	21.9	22.8	479; 569
	PEP-SC	546	21.9	23.1	501; 591
**TTS (s)**					
**Landing condition**	PP	0.948	0.022	20.7	0.90; 0.99
	UN-EC	0.952	0.022	20.7	0.91; 1.00
	UN-EI	0.938	0.022	20.9	0.89; 0.98
**Treatment**	PEP	0.937	0.022	20.7	0.89; 0.98
	PEP+	0.956	0.022	20.8	0.91; 1.00
	PEP-SC	0.945	0.022	20.9	0.90; 0.99

Abbreviation: pVGRF = peak vertical ground reaction force; CoP = center of pressure path length; TTS = time to stabilization; PP = pre-planned; UN-EC = unplanned expectation-consistent; UN-EI = unplanned expectation-inconsistent.

A significant main effect of treatment was found for time to pVGRF. However, this effect did not remain significant after Holm adjustment (Table 2). No significant treatment × landing condition interactions were detected for any outcome (all p > .05; Table 2). However, interaction plots (Fig 6A–D) descriptively suggested a trend toward slightly more favorable landing patterns after PEP + , with lower pVGRF, longer time to pVGRF, and lower CoP fluctuations (Fig 6A-C), particularly in the unplanned expectation-inconsistent condition. Higher conditional relative to marginal R^2^ values indicate substantial interindividual variability, with a large proportion of the explained variance attributable to between-subject differences across outcomes (Table 2).

**Fig 6:**
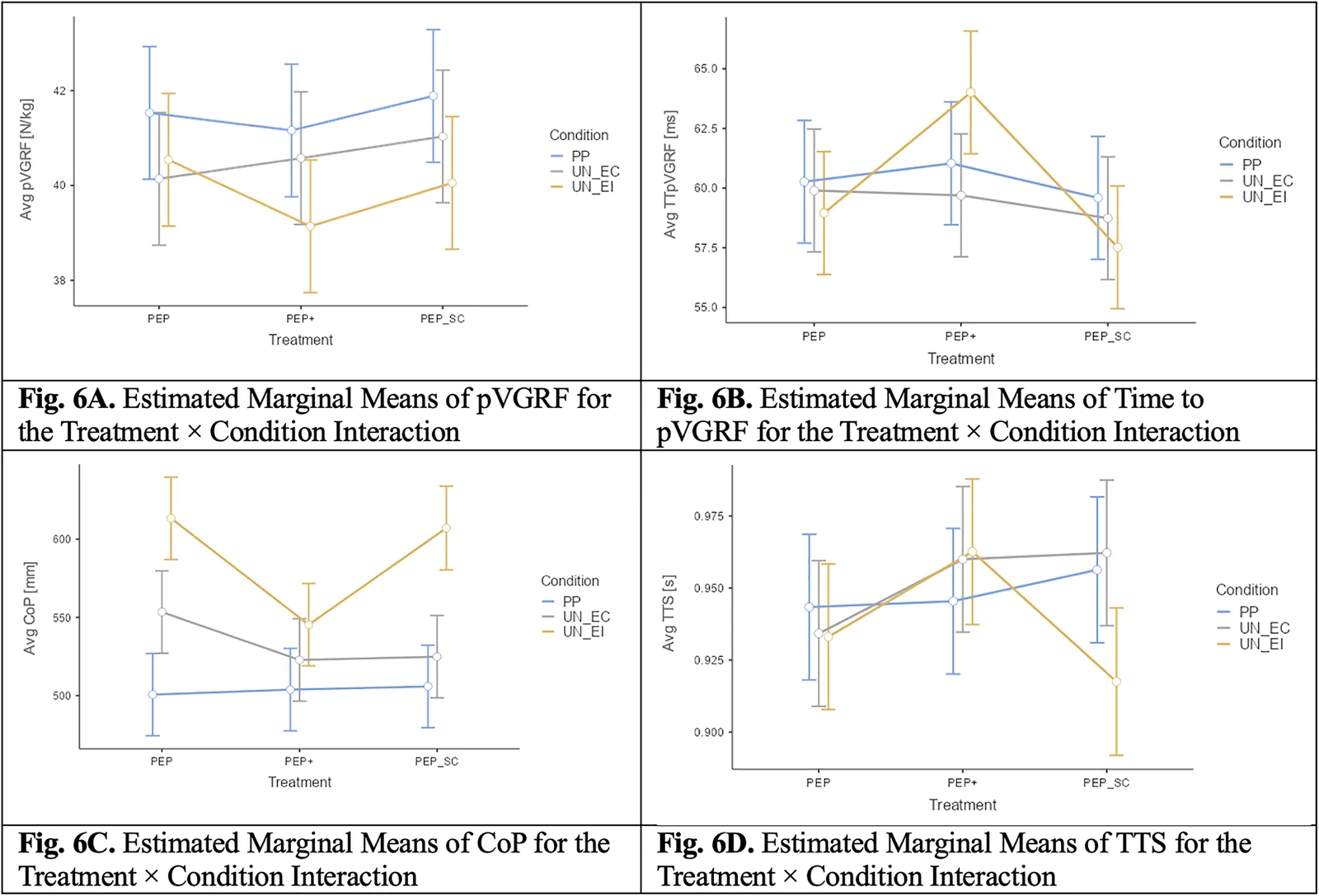
Estimated Marginal Means of the biomechanical outcomes pVGRF, Time to pVGRF, CoP, and TTS for treatment and condition. Abbreviations: pVGRF = peak vertical ground reaction force; TTpVGRF = time to pVGRF; CoP = center of pressure path length; TTS = time to stabilization; Avg = average; PP = pre-planned; UN-EC = unplanned expectation-consistent; UN-EI = unplanned expectation-inconsistent.

### Main fixed effects for error outcomes

For standing and decision error counts, significant main effects of landing condition were observed (standing errors: χ^2^ = 20.04, df = 2, p = .002; decision errors: χ^2^ = 53.79, df = 2, p = .002). Holm-adjusted post hoc tests indicated higher error counts in both unplanned conditions compared with the pre-planned condition, as well as higher errors in the unplanned expectation-inconsistent compared with the expectation-consistent condition (z = −2.28 to −5.76, p = .037 to <.001). Corresponding estimated marginal means are shown in Table 4. No significant main effects of treatment or treatment × landing condition interactions were observed (χ^2^ = 0.15–0.74, p = .742–.947).

**Table 4:** Estimated marginal means (expected counts) for standing and decision errors by Condition and Treatment (adjusted for period and average flight time).

Standing Errors (n)	Level	Mean	SE	95%-CI
Condition	PP	0.279	0.0817	0.16; 0.50
	UN-EC	0.527	0.1308	0.32; 0.86
	UN-EI	0.863	0.1937	0.56; 1.34
Treatment	PEP	0.477	0.124	0.29; 0.79
	PEP+	0.525	0.134	0.32; 0.87
	PEP-SC	0.506	0.130	0.31; 0.84
**Decision Errors (n)**				
Condition	PP	0.015	0.015	0.00; 0.11
	UN-EC	0.658	0.125	0.45; 0.95
	UN-EI	1.800	0.264	1.35; 2.40
Treatment	PEP	0.249	0.093	0.12; 0.52
	PEP+	0.283	0.105	0.14; 0.59
	PEP-SC	0.250	0.093	0.12; 0.52

Abbreviation: PP = pre-planned; UN-EC = unplanned expectation-consistent; UN-EI = unplanned expectation-inconsistent.

### Sensitivity analysis and pre-post differences

For time to pVGRF, exclusion of one influential participant resulted in non-significant main effects of landing condition (F = 1.26, p = .286) and treatment (F = 2.24, p = .110), while the interaction between these factors approached but did not reach statistical significance (F = 2.43, p = .051), with the descriptive pattern indicating longer time to pVGRF following PEP+ during the unplanned expectation-inconsistent condition. The additional inclusion of age, sex, and Tegner activity score as covariates did not meaningfully alter the main effects for any outcome.

Pre-post comparisons showed no significant differences between warm-up interventions for maximum CMJ height (F = 1.64, p = .209, η^2^ = 0.011) or Trail-Making-Test completion time F = 0.382, p = .685, η^2^ = 0.008).

## Discussion

The present study examined how the incorporation of specific cognitive and reactive agility demands into the standard PEP warm-up acutely affects jump-landing performance during pre-planned and unplanned single-leg landings in amateur team-sport athletes. The finding that the unplanned conditions resulted in altered dynamic landing control and lower landing success rates supported our first hypothesis regarding significant landing condition effects. Descriptive trends suggested more favorable landing performance outcomes after PEP+ during the unplanned expectation-inconsistent condition. However, after Holm adjustment, no significant treatment or treatment × landing condition interaction effects remained. Therefore, hypotheses 2 and 3 could not be confirmed. Consequently, these treatment-related findings should be considered exploratory and interpreted with caution.

### Landing condition effects

The unplanned conditions resulted in reduced landing stability, reflected by greater CoP fluctuations, and reduced landing quality, indicated by higher numbers of standing and decision errors compared with the pre-planned condition. Performance further deteriorated in the unplanned expectation-inconsistent condition relative to the expectation-consistent condition.

These findings may be likely explained by increased temporal and perceptual-cognitive demands during unplanned conditions. The maintenance of dynamic joint stability during jump landing is based on an optimally coordinated anticipatory (feedforward) and reflective (feedback) activation of the joint-stabilising musculature [50,51]. During unplanned conditions, however, the shorter available response time and increased perceptual-cognitive demands may limit the time available to prepare the landing and increase reliance on feedback control [13]. This reduced preparation may impair movement control during landing, resulting in less stable and more erroneous landings. While the expectation-consistent condition primarily adds uncertainty and attentional demands, the expectation-inconsistent condition additionally requires participants to inhibit the initial motor plan and rapidly adjust their movement when the landing side changes under severe time-constraints [32]. This likely contributed to the poorest landing performance observed in this condition and supports the proposed progressive increase in task complexity, reflecting team-sport-specific situations that require rapid movement adaptations to unexpected changes under time pressure [6].

This is in line with previous research reporting greater CoP fluctuations and higher numbers of erroneous landings during unplanned compared with pre-planned landing tasks [9,10,12], as well as altered joint-specific biomechanics, including increased knee abduction and tibial rotation moments and stiffer landing patterns, which may increase knee injury risk [13,14]. Similarly, Aflatounian et al. [52] reported reduced peak knee flexion during rapid decision-making, supporting stiffer landing patterns under increased cognitive demands.

Contrasting with this pattern, the unplanned expectation-inconsistent condition resulted in significantly lower pVGRF, which may reflect altered impact absorption strategies. This finding contrasts with previous research suggesting that unplanned landings are typically associated with stiffer landing strategies and higher impact forces [11,52–54], although null findings have also been reported [10,55]. While most previous studies did not distinguish between expectation-consistent and expectation-inconsistent unplanned conditions, significantly lower pVGRF values were observed only in the expectation-inconsistent condition, suggesting that the motor-cognitively most demanding condition may have induced a more cautious landing strategy. Similar landing adaptation patterns suggesting a “safety-first” strategy have been reported previously, indicating that unplanned conditions may elicit protective movement responses associated with lower measured vertical ground-reaction forces, potentially reflecting altered impact absorption strategies [15,32]. However, as the observed differences were small (~4% compared to the pre-planned conditon) and were not accompanied by shorter times to pVGRF, an important contributor to joint loading [56], these findings should be interpreted cautiously. In addition, differences in movement patterns between successful and erroneous trials may have influenced the observed pVGRF.

### Treatment and interaction effects

No significant main effects of treatment or treatment × landing condition interactions were observed for any outcome. However, descriptive trends suggested slightly more favorable landing patterns after the PEP+ program in the expectation-inconsistent unplanned condition. A significant main effect of treatment was initially observed for time to pVGRF, and sensitivity analysis excluding one influential participant revealed a treatment × landing condition interaction that approached statistical significance, favoring PEP+. However, these effects did not remain significant after Holm adjustment and should therefore be considered exploratory. Nevertheless, these trends may indicate that PEP+ facilitated more efficient cognitive processes (e.g., faster visuomotor processing or executive functions such as inhibitory control and decision-making), potentially supporting rapid movement adjustments. If PEP+ facilitated more efficient cognitive processing, the additional time available during the jump phase may have been used more effectively to support feedforward control and, consequently, landing stability and impact absorption. This interpretation is consistent with the lower CoP and pVGRF values and longer times to pVGRF, which may reflect altered global landing control and impact absorption strategies [56]. Although previous evidence suggests relationships between perceptual-cognitive and executive functions and safer biomechanical landing patterns under unplanned conditions [18,20], whether improved cognitive processing following PEP+ contributed to these descriptive trends remains speculative given the non-significant findings and the exploratory nature of the study. Limited statistical power and substantial interindividual variability may also have contributed to the absence of significant treatment and interaction effects.

A further explanation for the non-significant effects may be that the cognitive load, type, or dose of the PEP warm-up interventions was insufficient to elicit more robust effects on unplanned landing performance. This is in line with a previous randomized controlled trial [24], showing that visuomotor reaction training of the upper and lower extremities improved task-specific performance but did not transfer to unplanned landing outcomes, such as pVGRF, TTS, or decision error rates. These findings suggest that more complex or higher-order cognitive demands (e.g., executive functions) may be required to induce transfer to sport-specific movement tasks. In addition, acute motor-cognitive stepping exercises involving inhibition and decision-making during a 5-minute break following a fatigue protocol have been shown to enhance agility performance compared with a passive break [57], further supporting the role of higher cognitive demands during time-constrained reactive movement adaptations. This may also explain why no effects favoring the PEP-SC protocol were observed, despite the inclusion of dual-task agility exercises (multiple object tracking), which engage perceptual-cognitive and executive functions [58]. However, the overall dose of these cognitive demands may have been too low to induce meaningful acute effects. This is supported by the lower proportion of cognitive content in PEP-SC (25% vs. 50% in PEP+). Notably, descriptive trends in some outcomes were in favor of PEP+, suggesting that a higher proportion of cognitive stimulation may be relevant for eliciting acute motor-cognitive benefits. However, the absence of pre-post effects on Trail-Making-Test-B performance (reflecting executive functions such as working memory and cognitive flexibility; [59]) suggests that the overall cognitive demand may have been insufficient to induce measurable acute improvements in executive functions, which have been discussed as relevant in unplanned movement conditions [18,20].

### Practical implications and future research

Our findings of less stable landings and higher error rates during unplanned jump landings, particularly under expectation-inconsistent conditions, highlight the greater motor-cognitive demands imposed by time-constrained decisions and reactive responses. Practitioners such as athletic trainers and therapists may consider using the present jump-landing approach with graded task complexity to assess how athletes cope with such unplanned movement demands during performance or injury-risk screening contexts. Although not hazardous under controlled laboratory conditions, standing and decision errors may reflect team-sport-specific situations in which responses to external stimuli (e.g., approaching opponents) are delayed or inaccurate, such as incorrect foot placement (decision errors), or are executed under less stable conditions, as indicated by increased standing errors or greater CoP fluctuations [9]. This may be relevant, as impaired dynamic landing control and erroneous landings have been discussed as potential contributors to non-contact injury risk [9,37–41]. The task design is low-cost, easily reproducible, and does not require force plates or three-dimensional motion analysis systems. While only non-significant trends were observed for the PEP+ warm-up, its implementation requires no additional time compared with the standard PEP protocol and may represent a feasible approach for integrating motor-cognitive demands into warm-up routines.

Given the exploratory and non-significant findings regarding treatment effects, future studies with larger samples are needed to investigate the observed trends and determine whether motor-cognitively adapted warm-up protocols can improve unplanned landing performance before definitive recommendations can be made. Future research should also consider increasing the proportion of motor-cognitive demands and/or incorporating greater demands on executive functions. Beyond perceptual-cognitive and executive function demands, future studies may examine whether incorporating stroboscopic visual constraints, which challenge visual perception and anticipation, into agility-based warm-up components [60] influences subsequent unplanned landing performance. Such approaches may offer a potential strategy to target perceptual-cognitive and perception–action coupling demands relevant to reactive movement situations. Future research should examine whether motor-cognitively adapted warm-up protocols (e.g., the adapted PEP versions) influence joint-specific biomechanics related to injury risk (e.g., knee flexion angles or abduction moments), further assess the sensitivity of different biomechanical surrogates of dynamic landing control and load absorption to detect such changes, and evaluate the feasibility of implementing these protocols within regular training and match settings. In addition to jump-landing tasks, future studies investigating motor-cognitive warm-up effects should include cutting maneuvers, as these movements are highly relevant to injury mechanisms in team-sports [5]. Moreover, the effectiveness and applicability of such protocols warrant specific investigation in female athletes given their elevated risk for knee injuries, including ACL tears [61]. Future studies may also benefit from more homogeneous samples regarding sex, sport type, and performance level or from adequately powered subgroup analyses to reduce between-subject variability and better understand the potential effects of these adapted PEP protocols.

### Strengths and limitations

The absence of carryover, period or sequence effects suggests that the crossover design functioned as intended and supports the internal validity of the study. Additional strengths include controlling for flight time (jump height/available response time) and potential period effects related to learning and practice, as well as sensitivity analyses including sex, age, and Tegner activity score as covariates. These approaches accounted for relevant sources of variability and supported the robustness of the findings, but do not exclude limited power to detect smaller treatment or treatment × landing condition effects. Moreover, the absence of pre-post performance decrements in Trail-Making-Test-B time and maximum CMJ height suggests that the warm-ups did not induce measurable short-term impairments in executive function or explosive strength.

This study is not without limitations. First, a major limitation concerns the unequal type and dose of motor-cognitive demands across the adapted warm-up interventions. Although both PEP+ and PEP-SC included reactive agility components, albeit to a different extent, motor-cognitive exercises accounted for approximately 50% of the PEP+ session but only 25% of the PEP-SC session. Consequently, it remains unclear whether the descriptively more favorable trends following PEP+ were related to the greater dose, broader spectrum, or specific type of cognitive demands, limiting direct comparability between both interventions. Moreover, analyses of cognitive load and perceived exertion would have allowed a more comprehensive interpretation of the cognitive and physical demands of the warm-up interventions.

Second, the relatively small sample size may have reduced sensitivity for detecting smaller treatment and interaction effects, increasing the risk of type II error, particularly after adjustment for multiple comparisons. Therefore, the treatment-related findings should be considered exploratory and require confirmation in larger studies. Third, heterogeneous sport backgrounds (football, basketball, American football, tennis, volleyball) represent another limitation, as sport-specific cognitive and biomechanical demands, as well as sex-related differences in biomechanics and injury risk profiles [61], may have contributed to variability in outcomes and intervention responses.

Fourth, the jump-landing task did not directly assess established joint-specific biomechanical injury risk factors. Instead, the study relied on global surrogate measures of dynamic landing control and impact load absorption, which may be interpreted as functional indicators of overall movement control. These measures may not have been sufficiently sensitive to detect subtle motor-cognitive adaptations compared with more joint-specific biomechanical assessments. Finally, anticipatory guessing of the landing side may have influenced movement behavior and landing performance in some participants, particularly those with high error rates, with differences in successful trials potentially contributing to variability in landing performance.

## Conclusion

The present study investigated the acute effects of two motor-cognitively adapted warm-up interventions compared with the established neuromuscular PEP warm-up with no specific cognitive demands on pre-planned and unplanned jump-landings in amateur team-sport athletes. Unplanned landing conditions resulted in less stable landings and higher error rates than pre-planned landings. Although no significant treatment or treatment × landing condition interaction effects remained after Holm adjustment, exploratory trends suggested that the adapted PEP+ intervention may be associated with more favorable landing patterns regarding dynamic landing control and load absorption during unplanned landings. Future studies with larger, adequately powered samples are needed to determine whether these exploratory trends can be confirmed and to further examine potential effects on joint-specific biomechanics relevant to injury mechanisms.

## Supporting information

S1 Table Trial protocol. Overview of the methodology and deviations from the original study protocol.
(DOCX)

S1 File CONSORT checklist. CONSORT checklist of information about this randomized trial.
(DOCX)

S2 File Anonymized data sheet. Anonymized data sheet with relevant study data.
(XLSX)
