# Supplementary material for: Acute effects of motor-cognitive injury-prevention warm-ups on unplanned jump-landing performance in team-sport athletes: A randomized crossover trial
Source: PLoS One. 2026 Oct 9;21(10):e0360379. doi: 10.1371/journal.pone.0360379 (PMC13652156; doi:10.1371/journal.pone.0360379)
Supplement: Supplementary file 1 [file pone.0360379.s001.docx]

| **Methodological Category** | **Previus study** | **Current study** | **Deviation / Modification** |
| --- | --- | --- | --- |
| Study Design | Controlled 2-arm crossover | Randomized 3-arm crossover | Increased number of intervention arms |
| Ethics / Registration | Approved by Ethics Committee; DRKS00016942 | Approved by Ethics Committee; DRKS00016942 | No change |
| Participants | 22 healthy male amateur football players, 18–30 years | 21 healthy team-sport athletes, 18–30 years | No change |
| Recruitment | Flyers, social media, personal approach | Flyers, social media, personal approach | No change |
| Exclusion Criteria | Lower limb or head injuries, acute/chronic disease, substance use, muscle soreness | Lower limb or head injuries, acute/chronic disease, substance use, muscle soreness | No change |
| Sample Size / Power | Calculated based on 2-arm study, effect size f=0.52, target n=22 | Calculated based on 3-arm study, conservative f=0.4, target n=18 | Adjustment for new study design |
| Washout / Test Days | 2 test days, 3–7 days washout | 3 test days, ≥7 days washout | More test days for additional intervention arm |
| Randomization | Intervention order (2 arms) | Six possible sequences for 3 arms | More complex randomization |
| Warmup Interventions | PEP program, 10 min, 13 exercises in 4 categories (no static stretching, no "Russian Hamstring"); cycling ergometer warmup (control condition) | PEP program, 10 min, 12 exercises in 4 categories (no static stretching, no "Russian Hamstring") as control condition, plus two additional intervention arms: PEP with added cognitive tasks and PEP with added agility exercises | Same PEP warmup program as a foundation, with additional interventions incorporating cognitive and agility challenges; Lateral and forward/ backward hops were put together but doubled in time, results in same duration |
| Jump-Landing Task | 16 CMJs: preplanned (n=4) & unplanned (n=12), randomized | 22 CMJs: preplanned (n=6) & unplanned (n=16), randomized | Few more jumps for greater expressiveness |
| Equipment | Force plate (CSMi), HUMAC Balance System, 100 Hz | Capacitative pressure plate, 60 Hz | Minimal changes |
| Outcomes | pVGRF, TTpVGRF, CoP, TTS, errors | pVGRF, TTpVGRF, CoP, TTS, errors, cognitive and physical performance meassure | Minimal changes |
| Statistical Analysis | Parametric and non-parametric tests; carryover effects assessed via inter-individual sums; paired tests to evaluate treatment effects | Similar approach, adapted for three conditions using linear and generalized mixed models | Statistical analysis adapted to accommodate the three-arm crossover design |
